# The complete chloroplast genome of *Stauntonia chinensis* and compared analysis revealed adaptive evolution of subfamily Lardizabaloideae species in China

**DOI:** 10.1186/s12864-021-07484-7

**Published:** 2021-03-06

**Authors:** Feng Wen, Xiaozhu Wu, Tongjian Li, Mingliang Jia, Xinsheng Liu, Liang Liao

**Affiliations:** 1 School of Pharmacy and Life Science, Jiujiang University, Jiujiang, China; 2 State Key Laboratory of Ecological Pest Control for Fujian and Taiwan Crops, College of Plant Protection, Fujian Agriculture and Forestry University, Fuzhou, China

**Keywords:** Herbal medicine, Plastome, Adaptation, Positive selection, Phylogeny analyses

## Abstract

**Background:**

*Stauntonia chinensis* DC. belongs to subfamily Lardizabaloideae, which is widely grown throughout southern China. It has been used as a traditional herbal medicinal plant, which could synthesize a number of triterpenoid saponins with anticancer and anti-inflammatory activities. However, the wild resources of this species and its relatives were threatened by over-exploitation before the genetic diversity and evolutionary analysis were uncovered. Thus, the complete chloroplast genome sequences of *Stauntonia chinensis* and comparative analysis of chloroplast genomes of Lardizabaloideae species are necessary and crucial to understand the plastome evolution of this subfamily.

**Results:**

A series of analyses including genome structure, GC content, repeat structure, SSR component, nucleotide diversity and codon usage were performed by comparing chloroplast genomes of *Stauntonia chinensis* and its relatives. Although the chloroplast genomes of eight Lardizabaloideae plants were evolutionary conserved, the comparative analysis also showed several variation hotspots, which were considered as highly variable regions. Additionally, pairwise Ka/Ks analysis showed that most of the chloroplast genes of Lardizabaloideae species underwent purifying selection, whereas 25 chloroplast protein coding genes were identified with positive selection in this subfamily species by using branch-site model. Bayesian and ML phylogeny on CCG (complete chloroplast genome) and CDs (coding DNA sequences) produced a well-resolved phylogeny of Lardizabaloideae plastid lineages.

**Conclusions:**

This study enhanced the understanding of the evolution of Lardizabaloideae and its relatives. All the obtained genetic resources will facilitate future studies in DNA barcode, species discrimination, the intraspecific and interspecific variability and the phylogenetic relationships of subfamily Lardizabaloideae.

**Supplementary Information:**

The online version contains supplementary material available at 10.1186/s12864-021-07484-7.

## Background

Herbal medicine has been used as complementary and alternative treatments to augment existing therapies all over the world. The bioactive natural compounds extracted in herbal medicine may have the potential to form new drugs to treat a disease or other health conditions [1]. However, the wild resources of these plant species were on the verge of exhaustion by plundering exploitation with the increasing demand for herbal medicine with significant economic value [2]. Previous studies of herbal medicine species mainly concentrated on the cultivation and phytochemical studies. Whereas, few studies have described the genetic diversity and phylogenetic analysis. The germplasm, genetic and genomic resources need to be developed as potential tools to better exploit and utilize these herbal medicine species [3]. In addition, a good knowledge of genomic information of these species could provide insights for conservation and restoration efforts. Therefore, the molecular techniques are required to analyze the genetic diversity and phylogenetic relationship of these plants.

Chloroplasts contain their own genome, composing of approximately 130 genes, which has a typical quadripartite structure consisting of one large single copy region (LSC), one small single copy region (SSC) and a pair of inverted repeats (IRs) in most plants [4–6]. Unlike nuclear genomes, the chloroplast genome is a highly conserved circular DNA with stable genome, gene content, gene order, and much lower substitution rates [7–10]. Recently, with the development of next generation sequencing, it has become relatively easy to obtain the complete chloroplast genome of non-model taxa [11–13]. Thus, complete chloroplast genome has been shown to be useful in inferring evolutionary relationships at different taxonomic levels as an accessible genetic resource [14, 15]. On the other hand, although the chloroplast genome is often regarded as highly conserved, some mutation events and accelerated rates of evolution have been widely identified in particular genes or intergenic regions at taxonomic levels [7, 16–18]. The complete chloroplast genome has been considered to be informative for phylogenetic reconstruction and testing lineage-specific adaptive evolution of plants.

Lardizabaloideae (Lardizabalaceae) comprising approximately 50 species in nine genera [19]. It’s a core component of Ranunculales and belongs to the basal eudicots. Most species of Lardizabaloideae were considered as herbal medicinal plants, which were widespread in China, except tribe Lardizabaleae (including genus *Boquila* and genus *Lardizabala*). *Stauntonia chinensis* DC., belonging to the subfamily Lardizabaloideae, is widely grown throughout southern China, including Jiangxi, Guangdong, and Guangxi provinces [20]. It has been frequently utilized in traditional Chinese medicine known as “Ye Mu Gua” due to its anti-nociceptive, anti-inflammatory, and anti-hyperglycemic characteristics [21–23]. In this study, we reported and characterized the complete chloroplast genome sequence of *Stauntonia chinensis* and compared it with another 38 chloroplast genomes of Ranunculales taxa previously published (including species from Berberidaceae, Circaeasteraceae, Eupteleaceae, Lardizabalaceae, Menispermaceae, Papaveraceae, and Ranunculaceae). Our results will be useful as a resource for marker development, species discrimination, and the inference of phylogenetic relationships for family Lardizabalaceae based on these comprehensive analyses of chloroplast genomes.

## Results

### The chloroplast genome of *Stauntonia chinensis*

**Fig. 1:**
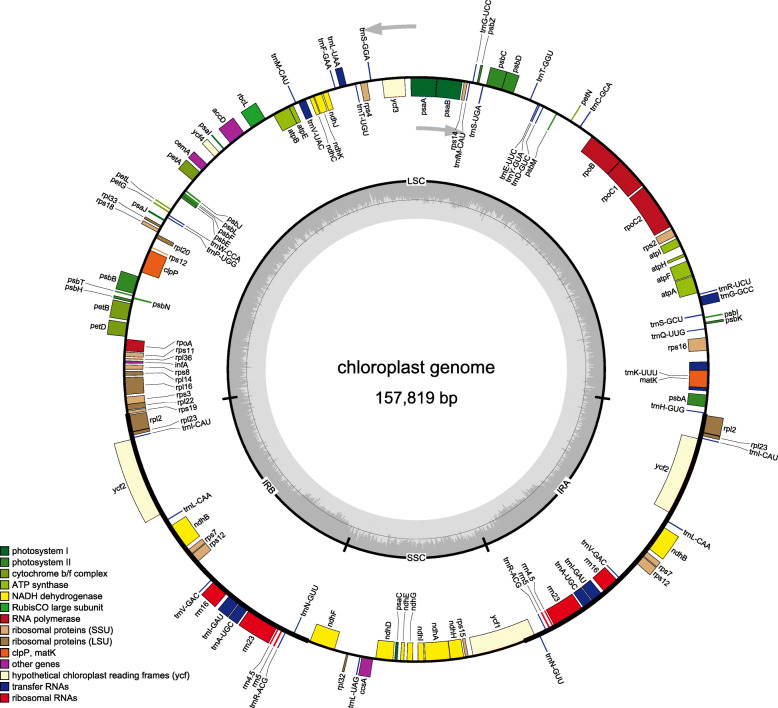
Gene map of the chloroplast genome of *Stauntonia chinensis*. Gray arrows indicate the direction of gene transcription. Genes belonging to different functional groups are marked in different colors. The darker gray columns in the inner circle correspond to the GC content, and small single copy (SSC), large single copy (LSC), and inverted repeats (IRA, IRB) are indicated respectively

We obtained 6.73 Gb of Illumina paired-end sequencing data from genomic DNA of *Stauntonia chinensis*. A total of 44,897,908 paired-end reads were retrieved with a sequence length of 150 bp, while a total of 41,809,601 of high-quality reads were used for mapping. The complete chloroplast DNA of *Stauntonia chinensis*. Was a circular molecule of 157,819 bp with typical quadripartite structure of angiosperms, which was composed of a pair of inverted repeats (IRA and IRB) of 26,143 bp each, separated by a large single copy (LSC) region of 86,545 bp and a small single copy (SSC) region of 18,988 bp (Fig. 1 and Table 1). The genome contained a total of 113 genes, including 79 unique protein-coding genes, 30 unique tRNA genes and 4 unique rRNA genes (Table 1). Of 113 genes, six protein-coding genes (*rpl2*, *rpl23*, *ycf2*, *ndhB*, *rps7*, and *rps12*), seven tRNA genes ((*trnI-CAU*, *trnL-CAA*, *trnV-GAC*, *trnI-GAU*, *trnA-UGC*, *trnR-ACG*, *trnN-GUU*) and 4 rRNA genes (*rrn16*, *rrn23*, *rrn4.5*, *rrn5*) were duplicated in the IR regions. The *Stauntonia chinensis* chloroplast genes encoded a variety of proteins, which were mostly involved in photosynthesis and other metabolic processes, including large rubisco subunit, thylakoid proteins and subunits of cytochrome b/f complex (Table 2). Among the *Stauntonia chinensis* chloroplast genes, fifteen distinctive genes, including *atpF*, *ndhA*, *ndhB*, *petB*, *petD*, *rpl2*, *rpl16*, *rpoC1*, *rps16*, *trnA-UGC*, *trnG-GCC*, *trnI-GAU*, *trnK-UUU*, *trnL-UAA*, and *trnV-UAC* harbored a single intron, and three genes (*clpP*, *rps12* and *ycf3*) contained two introns (Table 3). The gene *rps12* had trans-splicing, with the 5′-end exon 1 located in the LSC region and the 3′-exons 2 and 3 and intron located in the IR regions. The overall G/C content was 38.67%, whereas the corresponding values of LSC, SSC, and IR regions were 37.1, 33.68, and 43.08%, respectively.

**Table 1:** Statistics of the chloroplast genomes of *Stauntonia chinensis* and seven other Lardizabaloideae species

Species	Access No.	Genome length (bp)	GC content (%)	LSC length (bp)	SSC length (bp)	IR length (bp)	Gene Number	Protein-coding	tRNAs	rRNAs	No. of pseudogenes	GC3s content (%)
*Akebia trifoliata*	KU204898	158,339	38.7	87,057	19,024	26,129	132	85	37	8	2	28.5
*Akebia quinata*	KX611091	157,817	38.7	86,543	18,988	26,143	132	85	37	8	2	28.5
*Archakebia apetala*	MK468518	157,929	38.7	86,630	19,001	26,149	132	85	37	8	2	28.5
*Decaisnea insignis*	KY200671	158,683	38.5	87,187	19,162	26,167	132	85	37	8	2	28.3
*Holboellia angustifolia*	MN401677	157,797	38.7	86,543	18,972	26,141	132	85	37	8	2	28.5
*Holboellia latifolia*	MH394378	157,818	38.7	86,567	18,971	26,140	132	85	37	8	2	28.5
*Sinofranchetia chinensis*	MK533615	158,015	38.4	86,324	18,923	26,384	133	85	38	8	2	28.1
*Stauntonia chinensis*	MN401678	157,819	38.7	86,545	18,988	26,143	132	85	37	8	2	28.5

**Table 2:** Group of genes within the *Stauntonia chinensis* chloroplast genome

Group of genes	Gene names
Photosystem I	*psaA, psaB, psaC, psaI, psaJ*
Photosystem II	*psbA, psbB, psbC, psbD, psbE, psbF, psbH, psbI, psbJ, psbK, psbL, psbM, psbN, psbT, psbZ*
Cytochrome b/f complex	*petA, petB, petD, petG, petL, petN*
ATP synthase	*atpA, atpB, atpE, atpF, atpH, atpI*
NADP dehydrogenase	*ndhA, ndhB, ndhC, ndhD, ndhE, ndhF, ndhG, ndhH, ndhI, ndhJ, ndhK*
RubisCO large subunit	*rbcL*
RNA polymerase	*rpoA, rpoB, rpoC1, rpoC2*
Ribosomal proteins (SSU)	*rps2, rps3, rps4, rps7, rps8, rps11, rps12, rps14, rps15, rps16, rps18, rps19*
Ribosomal proteins (LSU)	*rpl2, rpl14, rpl16, rpl20, rpl22, rpl23, rpl32, rpl33, rpl36*
Hypothetical chloroplast reading frames(ycf)	*ycf1, ycf2, ycf3, ycf4*
Other genes	*accD, ccsA, cemA, clpP, infA, matK*
Ribosomal RNAs	*rrn4.5S, rrn5S, rrn16S, rrn23S*
Transfer RNAs	*trnA-UGC, trnC-GCA, trnD-GUC, trnE-UUC, trnF-GAA, trnfM-CAU, trnG-GCC, trnG-UCC, trnH-GUG, trnI-CAU, trnI-GAU, trnK-UUU, trnL-CAA, trnL-UAA, trnL-UAG, trnM-CAU, trnN-GUU, trnP-UGG, trnQ-UUG, trnR-ACG, trnR-UCU, trnS-GCU, trnS-GGA, trnS-UGA, trnT-GGU, trnT-UGU, trnV-GAC, trnV-UAC, trnW-CCA, trnY-GUA*

**Table 3:** Genes with introns in the chloroplast genome of *Stauntonia chinensis*

Gene	Location	Exon I (bp)	Intron I (bp)	Exon II (bp)	Intron II (bp)	Exon III (bp)
*trnK-UUU*	LSC	37	2476	35		
*rps16*	LSC	40	874	227		
*trnG-UCC*	LSC	23	712	49		
*atpF*	LSC	145	1088	71		
*rpoC1*	LSC	432	764	1614		
*ycf3*	LSC	124	721	230	737	153
*trnL-UAA*	LSC	35	508	50		
*trnV-UAC*	LSC	39	587	35		
*clpP*	LSC	71	798	291	653	247
*petB*	LSC	6	807	642		
*petD*	LSC	8	709	475		
*rpl16*	LSC	9	1102	399		
*rpl2*	IR	391	664	434		
*ndhB*	IR	777	696	756		
*rps12*^*a*^	IR	114		232	659	23
*trnI-GAU*	IR	37	939	35		
*trnA-UGC*	IR	38	800	35		
*ndhA*	SSC	553	1086	539		

^a^rps12 gene is trans-spliced gene with the two duplicated 3′ end exons in IR regions and 5′ end exon in the LSC region

### Codon usage bias pattern

**Fig. 2:**
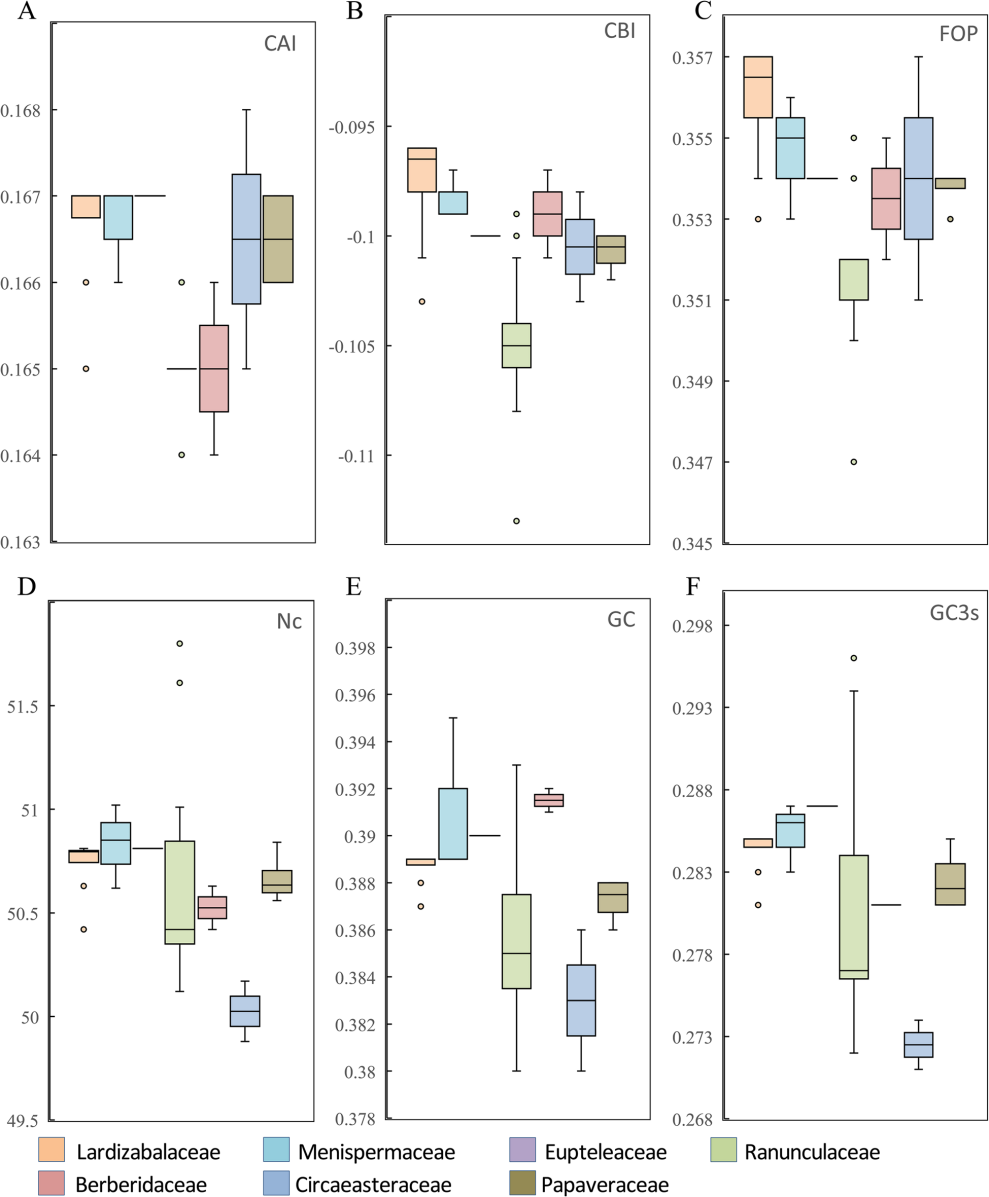
Statistics of codon usage bias in Lardizabaloideae and other family species. **a** CAI (Codon adaptation index), **b** CBI (Codon bias index), **c** FOP (Frequency of optimal codons index), **d** NC (Effective number of codons), **e** GC (GC content), **f** GC3s (GC of synonymous codons in 3rd position)

It is generally acknowledged that codon usage frequencies varied among genomes, among genes, and within genes [24]. Codon preferences was often explained by a balance between mutational biases and natural selection for translational optimization [25–27]. Optimal codons help to increase both the efficiency and accuracy of translation [28]. The codon usage and relative synonymous codon usage (RSCU) values in the *Stauntonia chinensis* chloroplast genome was calculated based on protein-coding genes (Table 4). In total, 85 protein-coding genes in the *Stauntonia chinensis* chloroplast genome were encoded by 26,246 codons. Among the codons, the most frequent amino acid was leucine (2701 codons, 10.29%), while cysteine (310 codons, 1.18%) was the least abundant amino acid excluding the stop codons. Similar to other angiosperm chloroplast genome, codon usage in the *Stauntonia chinensis* chloroplast genome was biased towards A and U at the third codon position, according to RSCU values (with a threshold of RSCU > 1) [29]. Further, the pattern of codon usage bias in the subfamily Lardizabaloideae and other species in Ranunculales were investigated (Fig. 2, Additional file 1). We found that two parameters (codon bias index, CBI and frequency of optimal codons, Fop) involved in codon usage bias were higher in Lardizabaloideae species than other species in Ranunculales.

**Table 4:** Relative synonymous codon usage (RSCU) in the *Stauntonia chinensis* chloroplast genome

Codon	Amino acid	Count	RSCU	Codon	Amino acid	Count	RSCU
UUU	F	849	1.18	UAU	Y	766	1.61
UUC	F	587	0.82	UAC	Y	185	0.39
UUA	L	743	1.65	UAA	*	38	1.34
UUG	L	570	1.27	UAG	*	25	0.88
CUU	L	586	1.3	CAU	H	507	1.51
CUC	L	206	0.46	CAC	H	164	0.49
CUA	L	383	0.85	CAA	Q	688	1.48
CUG	L	213	0.47	CAG	Q	239	0.52
AUU	I	1055	1.42	AAU	N	945	1.52
AUC	I	487	0.66	AAC	N	297	0.48
AUA	I	682	0.92	AAA	K	956	1.43
AUG	M	627	1	AAG	K	379	0.57
GUU	V	521	1.44	GAU	D	865	1.57
GUC	V	179	0.49	GAC	D	237	0.43
GUA	V	521	1.44	GAA	E	977	1.45
GUG	V	227	0.63	GAG	E	366	0.55
UCU	S	543	1.57	UGU	C	219	1.41
UCC	S	361	1.04	UGC	C	91	0.59
UCA	S	443	1.28	UGA	*	22	0.78
UCG	S	211	0.61	UGG	W	455	1
CCU	P	419	1.52	CGU	R	360	1.33
CCC	P	220	0.8	CGC	R	100	0.37
CCA	P	330	1.2	CGA	R	363	1.34
CCG	P	134	0.49	CGG	R	118	0.43
ACU	T	521	1.53	AGU	S	390	1.13
ACC	T	261	0.76	AGC	S	125	0.36
ACA	T	422	1.24	AGA	R	499	1.84
ACG	T	161	0.47	AGG	R	188	0.69
GCU	A	630	1.8	GGU	G	608	1.34
GCC	A	213	0.61	GGC	G	179	0.39
GCA	A	400	1.14	GGA	G	734	1.62
GCG	A	160	0.46	GGG	G	296	0.65

### Repeats and microsatellites analyses

**Fig. 3:**
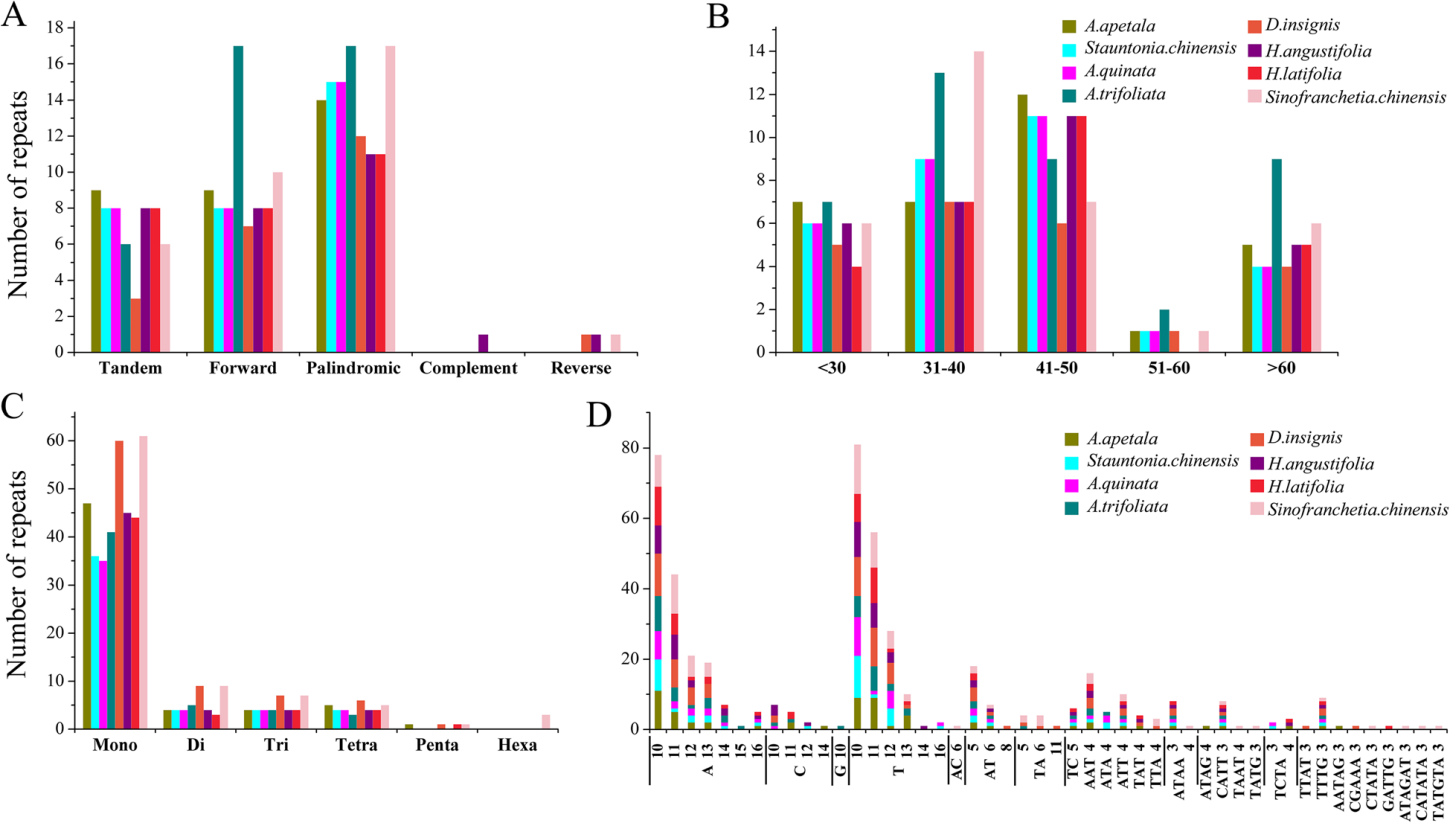
The repeat elements in the chloroplast genome of eight Lardizabaloideae species. **a** Number of five repeat types; **b** Number of repeat sequences by length; **c** Number of six SSR types; **d** Number of identified SSR motifs

Five type of repeat structures, including tandem, forward, palindromic, complement, and reverse repeats were identified using REPuter software in eight sequenced chloroplast genomes of Lardizabaloideae species. Overall, 23–40 repeat sequences were identified in each chloroplast genome, of which 3–9 tandem repeats, 7–17 forward repeats, and 11–17 palindromic repeats were separately detected, while few complement and reverse repeats were screened, for instance, only one complement repeat was predicted in *Holboellia angustifolia* (Fig. 3a). More than half of these repeats (72.5% at least) had a repeat length between 30 and 50 bp (Fig. 3b), and majority of the repeats were distributed in non-coding regions, including the intergenic regions and introns. Nevertheless, a small number of coding genes and tRNA genes were also found to contain repeat sequences, such as *ycf2*, *psaA*, *psaB*, *trnG* and *trnS* in *Stauntonia chinensis* chloroplast genome.

A total of 47–83 microsatellites were predicted in these eight chloroplast genomes, and the most predominant type of the SSRs were mononucleotides SSRs (especially for A/T, Fig. 3c). Besides, di-nucleotides were also detected in each chloroplast genomes, especially for AT5 and AT6. Furthermore, *Stauntonia chinensis* chloroplast genome contained four tri-nucleotides and four tetra-nucleotides, while other seven chloroplast genomes were found to have 34 tri-nucleotides and 31 tetra-nucleotides. Additionally, none of penta- and hexa-nucleotides were found in *Stauntonia chinensis* chloroplast genome. Similarly, SSRs mainly located in non-coding regions, particularly in intergenic regions, while several coding genes and tRNA genes such as *trnK*, *trnG*, *ycf3*, *trnL*, *ndhK*, *cemA*, and *ycf1* were also found to contain SSRs, especially, *ycf1* has three types of SSRs.

### Genome comparison

**Fig. 4:**
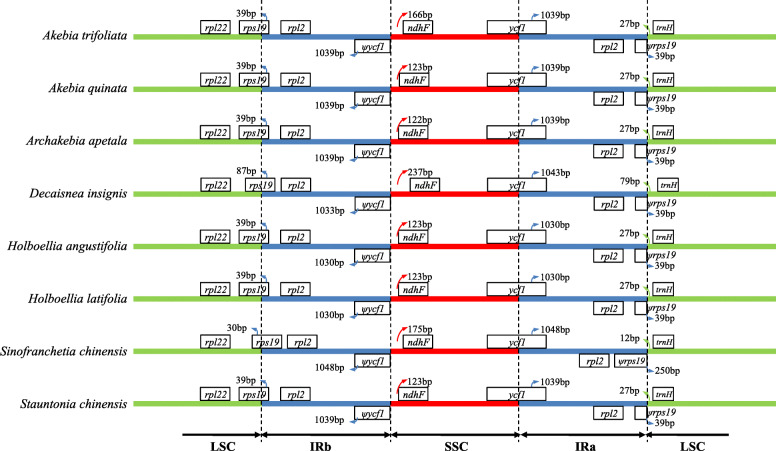
The comparison of the LSC, IR, and SSC boundary regions among the eight Lardizabaloideae species chloroplast genomes. φ indicates a pseudogene

The border regions and adjacent genes of chloroplast genomes were compared to analyze the expansion and contraction variation in junction regions, which were common phenomenons in the evolutionary history of land plants. To evaluate the potential impact of the junction changes, we compared the IR boundaries of the Lardizabaloideae species (Fig. 4). Although the majority of genomic structure, such as gene order and gene number were conserved, the eight chloroplast genomes of Lardizabaloideae species showed visible divergences at the IRA/LSC and IRB/SSC borders. Some differences in the IR expansions and contractions still existed. For example, the IRB region expanded into the gene *rps19* with 87 and 250 bp in the IRB regions of *Decaisnea insignis* and *Sinofranchetia chinensis* chloroplast genomes, respectively, although the IRB regions of other six chloroplast genomes were conserved. Thus, we found that the IR regions of the eight chloroplast genomes were conserved, except the chloroplast genomes of *Decaisnea insignis* and *Sinofranchetia chinensis,* which were slightly expanded compared with that of the other species.

**Fig. 5:**
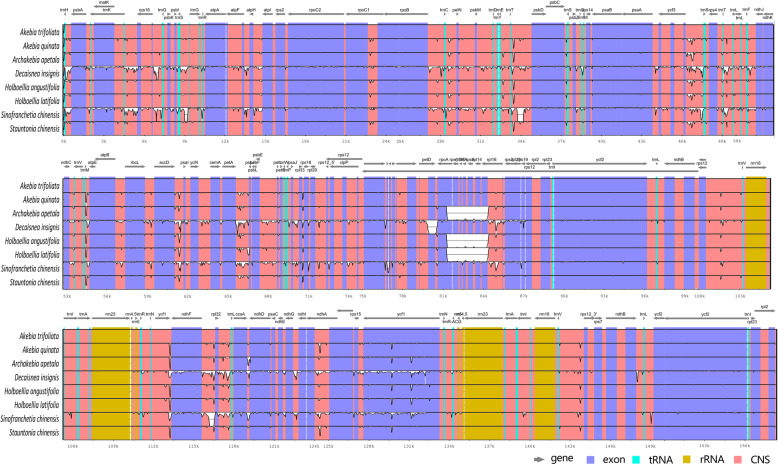
Sequence alignment of eight Lardizabaloideae chloroplast genomes using the mVISTA program with *A. trifoliata* as a reference. The y-axis represents the percent identity within 50–100%. The transcriptional direction of genes indicated by grey arrows. Genome regions are color-coded as protein-coding (exon), tRNA, rRNA, and conserved non-coding sequences (CNS)

**Fig. 6:**
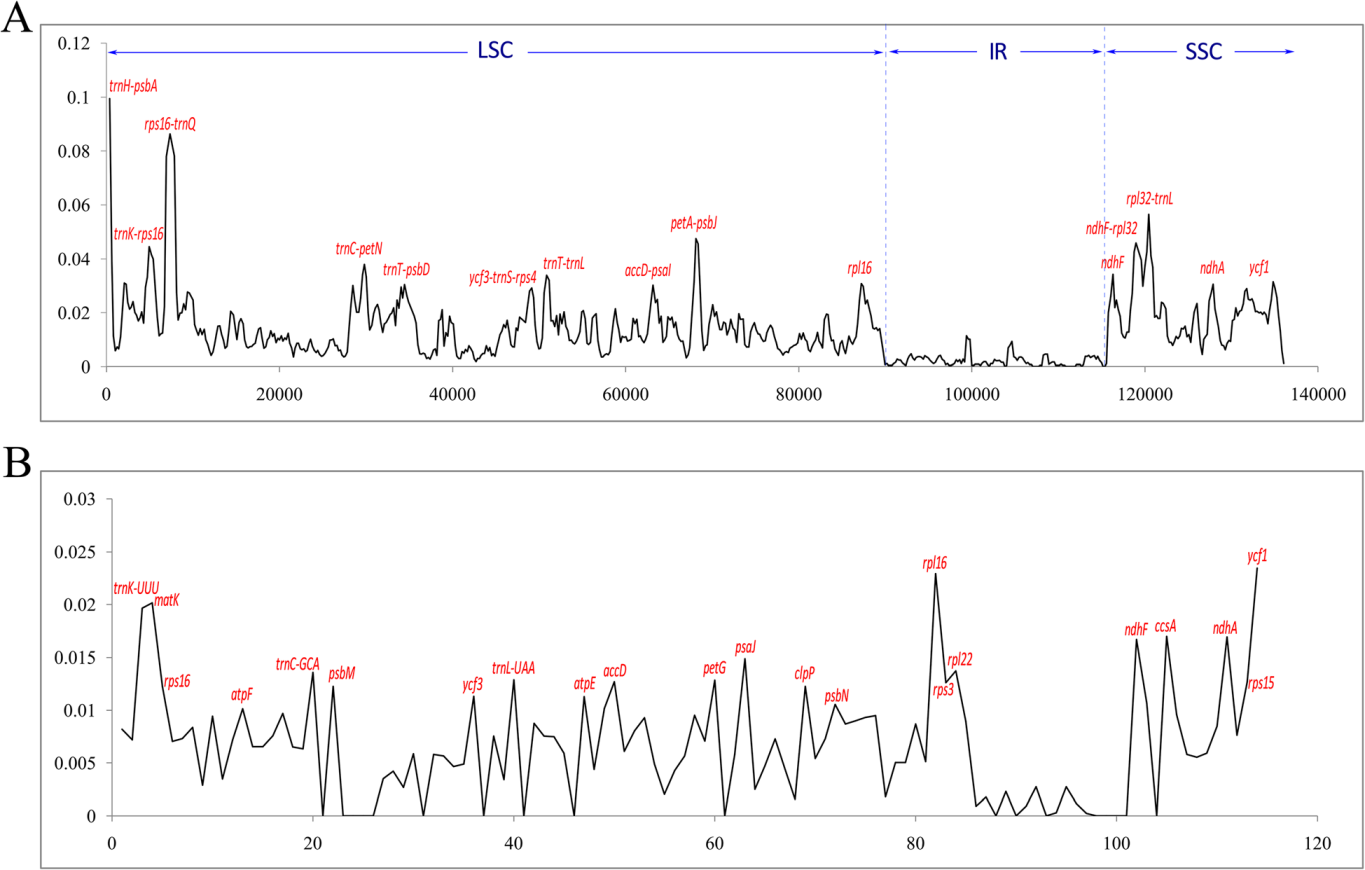
Nucleotide diversity (Pi) in the complete chloroplast genomes of eight Lardizabaloideae species. **a** Sliding window analysis with a window length of 600 bp and a step size of 200 bp. **b** The nucleotide diversity of chloroplast genes

To further investigate the divergence of chloroplast genomes among Lardizabaloideae species, a global sequence alignment of eight chloroplast genomes were compared using the annotated chloroplast genome of *A. trifoliata* as a reference. These closely related species had little difference in genome size, ranging from 157,797 bp to 158,683 bp. Although sequence similarities were very high in IR regions, the chloroplast genomes exhibited less conserved in LSC and SSC regions (Fig. 5). A sliding window analyses of the whole chloroplast genomes of eight Lardizabaloideae species indicated that most of the variation occurred in the LSC and SSC regions, which exhibited higher nucleotide variability (Pi) in comparison to IR regions (Fig. 6a). As shown in Fig. 6a, the nucleotide diversity values in the LSC and SSC regions ranged from 0.00173 to 0.08625 and from 0.0044 to 0.05637, respectively, while the value was from 0.00 to 0.01131 in the IRs regions. Expectedly, the divergence in intergenic regions was higher than in genic regions, but the *ycf1* gene exhibited a higher variability. The most divergent non-coding regions among the eight Lardizabaloideae chloroplast genomes were *trnH-psbA*, *trnK-rps16*, *rps16-trnQ*, *trnC-petN*, *trnT-psbD*, *ycf3-trnS-rps4*, *trnT-trnL*, *accD-psaI*, *petA-psbJ*, *ndhF-rpl32*, and *rpl32-trnL*. Although coding regions were conserved, minor sequence variation was observed among the eight chloroplast genomes in the *trnK*, *matK*, *psaJ*, *rpl16*, *ndhF*, *ccsA*, *ndhA*, and *ycf1* gene as shown in Fig. 6b (Pi value > 0.015). Similarly, mauve alignment results revealed that no large structural changes such as gene order rearrangements was detected across these eight chloroplast genomes of Lardizabaloideae species (Additional file 2), although some inversions were present in LSC and SSC regions in other Ranunculales species, such as *Pulsatilla chinensis*, *Anemone trullifolia*, and *Anemoclema glaucifolium*.

### Estimating rates of chloroplast evolution and positive selection analyses

**Fig. 7:**
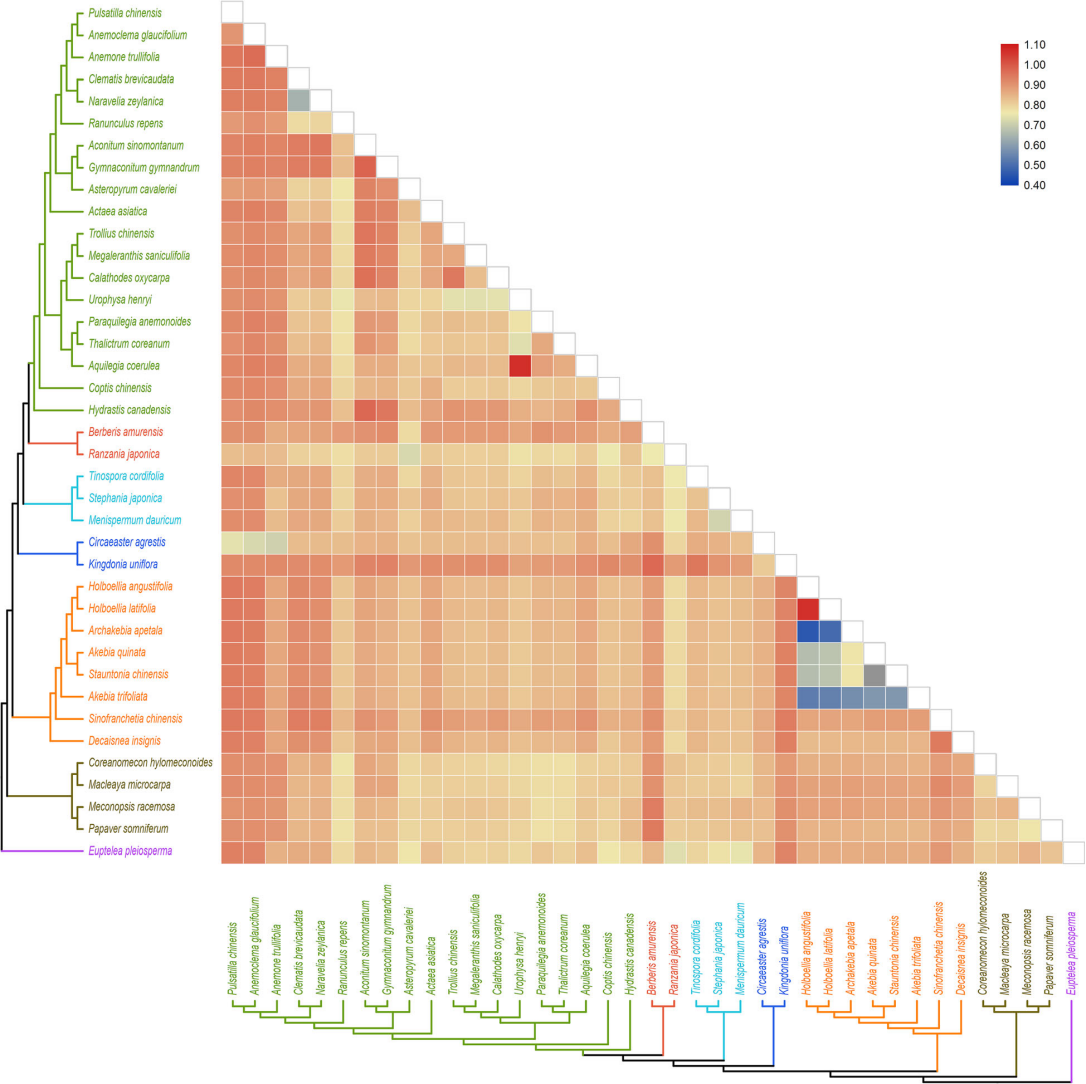
Pairwise Ka/Ks ratios in Lardizabaloideae and other families in order Ranunculales. Heatmap shows pairwise Ka/Ks ratios between every concatenated single-copy CDs sequence in the multigene nucleotide alignment

Most of Ka/Ks values of these Ranunculales species were less than or close to 1, providing the evidence that these chloroplast genes experienced purifying or no selection pressures (Fig. 7 and Additional file 3). Furthermore, in Lardizabaloideae species, the Ka/Ks ratios were far less than 1 among *Akebia trifoliata*, *Akebia quinata*, *Stauntonia chinensis*, and *Archakebia apetala*. However, the Ka/Ks ratio between *Holboellia angustifolia* and *Holboellia latifolia* was greater than 1, implying some chloroplast coding sites of these two species were under positive selection.

**Fig. 8:**
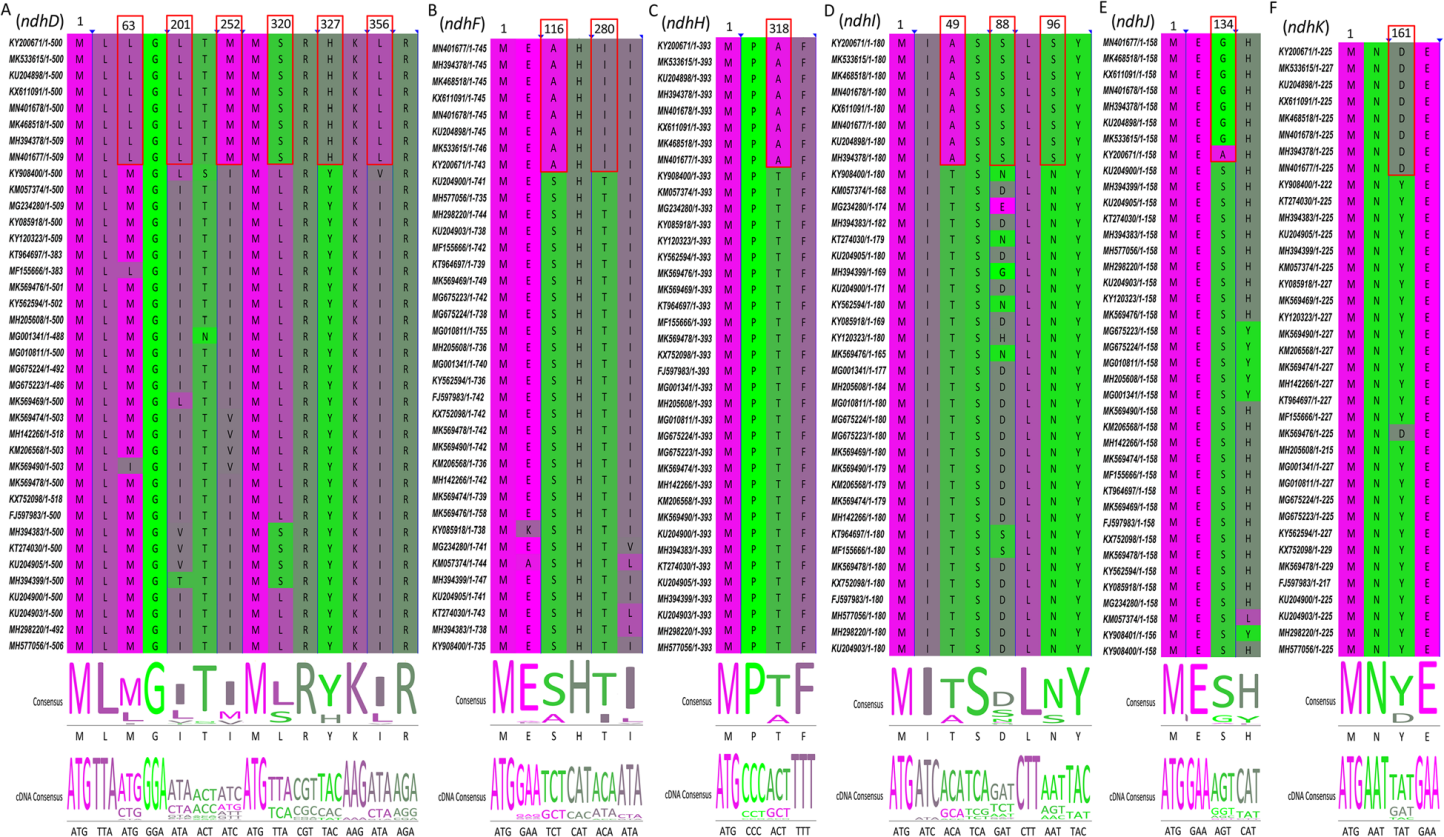
Partial alignment of six out of 25 positively selected genes. **a-f** Partial aligned amino acids sequences of the *ndhD*, *ndhF*, *ndhH*, *ndhI*, *ndhJ* and *ndhK* gene, respectively. The red blocks stand for the amino acids in Lardizabaloideae with a high BEB posterior probability

To further identify chloroplast protein-coding genes that might have undergone positive selection in Lardizabaloideae species, branch-site model analysis was employed by defining Lardizabaloideae species as foreground branch. A total number of 55 single-copy coding genes were considered for the positive selection analysis (Table 5). Although the likelihood ratio test showed that most of *p*-values were not significant in each gene range, two protein coding genes (*rbcL* and *accD*) indicated rejection of a null model (*p* < 0.05), corroborating the hypothesis that some amino acid sites in these two proteins in clade Lardizabaloideae species have been under positive selection (Table 5). Further analysis using a Bayes empirical Bayes (BEB) procedure identified 25 protein coding genes (*accD*, *atpA*, *atpE*, *atpI*, *ccsA*, *clpP*, *ndhD*, *ndhF*, *ndhH*, *ndhI*, *ndhJ*, *ndhK*, *psaA*, *psaB*, *psaI*, *psbA*, *psbZ*, *rbcL*, *rpl33*, *rpoA*, *rpoB*, *rpoC1*, *rps14*, *rps2*, and *ycf3*) with significant posterior probabilities suggesting some sites in these genes were under positive selection (Table 5, Fig. 8 and Additional file 4). Among them, 11 genes only had one positively selected site, whereas *accD* gene contained the largest number of positively selected sites (16 sites). Notably, most of *ndh* family genes possessed at least one positively selected site, implying this family members were potentially under positive selective pressure in Lardizabaloideae species (Fig. 8).

**Table 5:** The potential positive selection test based on the branch-site model

Gene	Null hypothesis			Alternative hypothesis			Significance test	
name	lnL	df	Omega (w = 1)	lnL	df	Omega (w > 1)	BEB	*P*-value
***accD***	− 6244.2703	76	1	− 6251.82	75	40.32412	78 I 0.946,79 D 0.840,80 S 0.573,81 G 0.779,82 K 0.969*,87 G 0.750,90 D 0.597,148 M 0.565,157 H 0.557,162 P 0.654,167 I 0.715,188 N 0.626,208 S 0.753,262 N 0.683,295 N 0.585,314 V 0.694	1.01E-04
***atpA***	− 7235.2876	80	1	−7235.29	79	1	123 V 0.784	9.99E-01
*atpB*	− 6617.3383	80	1	−6617.34	79	1.00000		1.00E+ 00
***atpE***	−2016.8158	80	1	−2018	79	87.46955	35 P 0.917,52 Y 0.977*	1.23E-01
*atpF*	− 857.07265	80	1	−857.073	79	1.00000		1.00E+ 00
*atpH*	− 800.09979	76	1	−800.1	75	3.48674		9.98E-01
***atpI***	− 3183.0473	80	1	−3183.51	79	20.22025	35 V 0.753,59 R 0.824,96 P 0.843,202 V 0.828	3.38E-01
***ccsA***	− 5988.5949	80	1	− 5989.75	79	15.32850	14 S 0.673,120 I 0.887	1.28E-01
*cemA*	− 4097.1783	79	1	−4097.63	78	999.00000		3.41E-01
***clpP***	− 3779.5729	78	1	−3779.64	77	1.73510	16 D 0.577,80 F 0.508,88 T 0.606,93 V 0.593,95 I 0.548	7.05E-01
*matK*	− 9319.3607	80	1	−9319.36	79	1.00000		1.00E+ 00
*ndhA*	− 5632.3781	78	1	−5632.38	77	1.00000		1.00E+ 00
*ndhC*	− 1481.7425	79	1	−1481.74	78	1.00000		9.99E-01
***ndhD***	− 6748.038	78	1	−6748.06	77	1.50673	63 M 0.697,201 I 0.662,252 I 0.664,320 L 0.685,327 Y 0.714,356 I 0.712	8.29E-01
*ndhE*	− 1550.4989	80	1	−1550.5	79	1.00000		1.00E+ 00
***ndhF***	−14,465.344	76	1	−14,465.3	75	1.00000	116 A 0.557,280 I 0.550	1.00E+ 00
*ndhG*	− 2862.0535	79	1	−2862.05	78	1.00000		1.00E+ 00
***ndhH***	− 5898.3248	78	1	−5898.32	77	1.00000	318 T 0.527	1.00E+ 00
***ndhI***	− 2246.7819	78	1	− 2247.13	77	13.49799	49 T 0.845,88 D 0.585,96 N 0.815	4.02E-01
***ndhJ***	− 2064.866	80	1	−2064.87	79	1.00000	134 G 0.745	1.00E+ 00
***ndhK***	− 2624.0218	76	1	−2624.02	75	1.00000	161 Y 0.548	1.00E+ 00
*petA*	− 2607.1562	80	1	−2607.16	79	10.70011		9.89E-01
*petG*	− 404.32829	80	1	−404.369	79	1.00000		7.76E-01
*petL*	− 386.63805	80	1	− 385.507	79	1.41918		1.33E-01
*petN*	−218.82071	80	1	−218.821	79	3.96657		9.95E-01
***psaA***	− 7414.7714	80	1	−7414.77	79	1.15065	441 V 0.583	9.85E-01
***psaB***	− 7487.909	80	1	−7487.91	79	1.00000	177 L 0.522	9.86E-01
*psaC*	−965.2544	80	1	−965.254	79	1.00000		9.99E-01
***psaI***	−489.20384	80	1	−489.204	79	1.00000	22 A 0.857	9.94E-01
*psaJ*	− 623.17909	80	1	− 622.516	79	1.00000		2.50E-01
***psbA***	−3805.4481	80	1	−3805.45	79	1.00000	3 A 0.784,228 T 0.784,350 S 0.738	1.00E+ 00
*psbC*	− 5464.4744	80	1	−5464.47	79	1.00000		1.00E+ 00
*psbD*	− 3430.3571	80	1	−3430.36	79	1.00000		9.89E-01
*psbE*	−824.21551	80	1	−824.216	79	1.00000		1.00E+ 00
*psbF*	− 297.73519	80	1	−297.735	79	1.00000		1.00E+ 00
*psbI*	−386.8387	80	1	−386.839	79	1.00000		1.00E+ 00
*psbJ*	−409.58353	80	1	−409.584	79	3.05096		9.99E-01
*psbK*	− 808.36136	80	1	−808.361	79	1.00000		1.00E+ 00
*psbL*	−251.14656	80	1	−250.996	79	1.00000		5.83E-01
*psbM*	−344.18423	80	1	−344.184	79	1.00000		1.00E+ 00
*psbN*	−339.98529	78	1	−339.985	77	3.88417		1.00E+ 00
***psbZ***	−615.4539	76	1	−615.454	75	1.00000	17 L 0.553	1.00E+ 00
***rbcL***	− 6385.4416	80	1	− 6388.64	79	999.00000	365 L 0.908	1.14E-02
*rpl20*	− 2104.3884	80	1	−2104.39	79	1.00000		1.00E+ 00
***rpl33***	− 895.54775	80	1	−895.771	79	84.08315	57 G 0.873	5.04E-01
***rpoA***	− 5786.0327	76	1	−5786.03	75	1.00000	5 A 0.523,107 Y 0.502,125 S 0.554,135 Q 0.559,276 K 0.555	1.00E+ 00
***rpoB***	−14,722.031	80	1	−14,722	79	1.24476	281 A 0.535,454 Y 0.537	9.55E-01
***rpoC1***	− 9096.3909	78	1	−9096.39	77	1.00000	110 A 0.561,590 P 0.569	1.00E+ 00
*rpoC2*	−21,869.149	80	1	−21,869.1	79	1.00000		9.99E-01
***rps14***	− 1369.7427	78	1	− 1370.22	77	999.00000	28 R 0.844,35 L 0.559	3.29E-01
*rps15*	−324.00056	74	1	−323.269	73	2.04455		2.26E-01
*rps18*	− 1134.1501	80	1	−1134.15	79	1.00000		1.00E+ 00
***rps2***	− 3349.317	80	1	−3349.32	79	1.00000	124 E 0.631,182 I 0.648	1.00E+ 00
***ycf3***	− 1601.2056	78	1	−1601.35	77	24.41198	5 R 0.733	5.89E-01
*ycf4*	− 2660.8473	79	1	− 2661.13	78	71.02868		4.55E-01

*Indicate that the posterior probabilities of the site is > 0.95

### Phylogenetic analyses

**Fig. 9:**
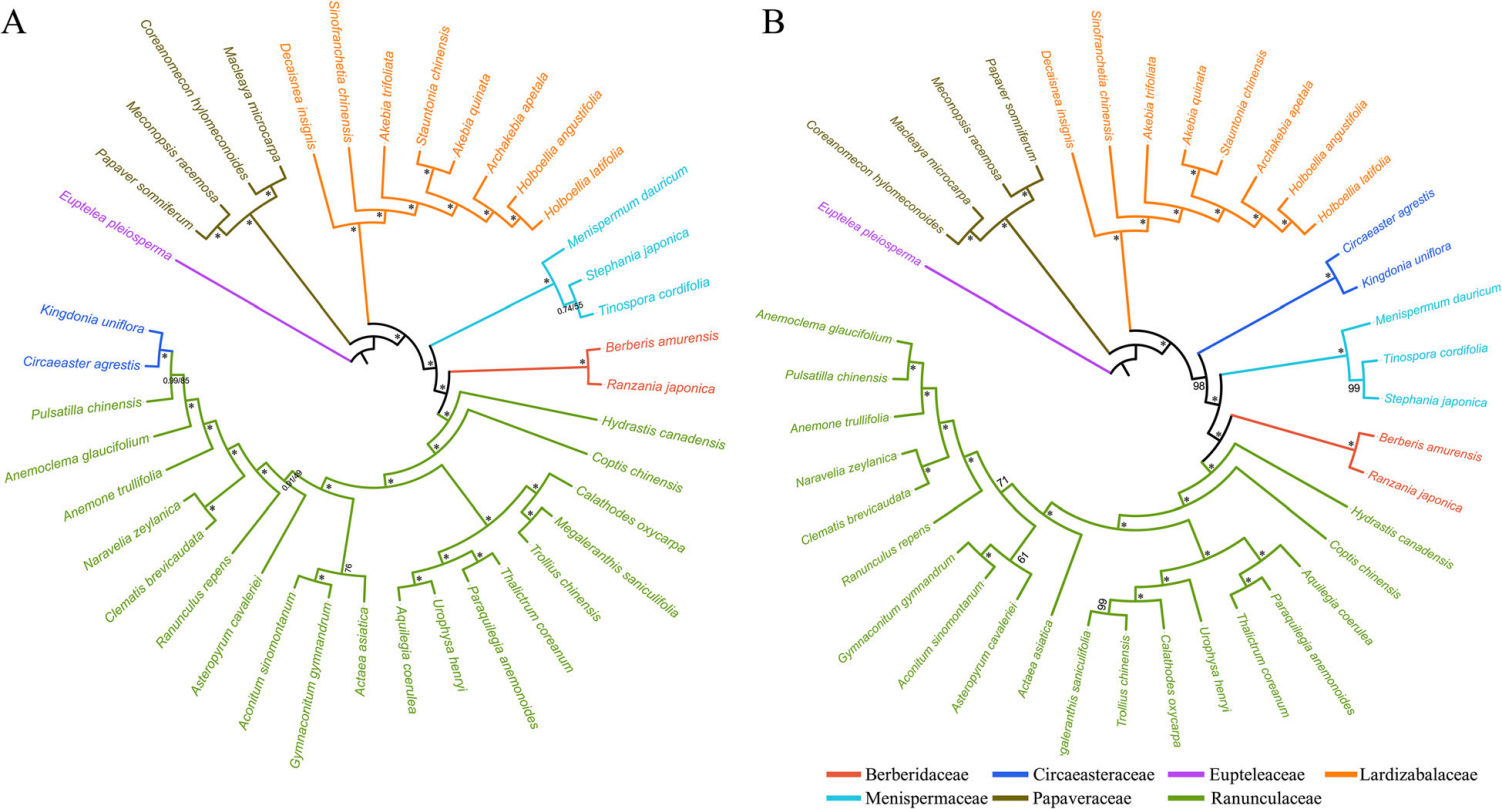
Phylogenetic relationships of Lardizabaloideae and closely related species in order Ranunculales. Tree constructed by Maximum likelihood (ML) and Bayesian inference (BI) methods with the posterior probabilities of BI and the bootstrap values of ML above the branches based on: **a** protein-coding genes CDs sequences, **b** the complete chloroplast genome sequences. * Represent maximum support in all two analyses

Bayesian and ML trees reconstructed based on the CCG dataset were highly congruent in identifying the phylogenetic position of these seven families in the order Ranunculales (Fig. 9). All nodes of these phylogenetic trees were strongly supported by bootstrap values (BS) in ML analysis and posterior probabilities (PP) in Bayesian analysis. The 39 taxa were classified into five major clades, of which Berberidaceae, Menispermaceae, and Ranunculaceae species clustered into a clade showed a close genetic relationship, while other family species constituted a monophyly. However, the family Circaeasteraceae species showed different position relative to other six families in Bayesian and ML reconstructed trees based on the protein-coding genes CDs dataset. The family Circaeasteraceae species were clustered into a clade with family Ranunculaceae species in phylogenetic tree based on CDs dataset, indicating that Circaeasteraceae had strong support to be a sister to the Ranunculaceae.

## Discussion

### Architecture of chloroplast genomes in subfamily Lardizabaloideae

Recently, chloroplast genomes have become to be useful tools to evaluate the genetic divergence among related species [30, 31]. Here we present the complete chloroplast genome of *Stauntonia chinensis*. The organization of the chloroplast genomes among eight Lardizabaloideae species exhibited a high degree of synteny, implying that these genomes were evolutionary conserved at the genome-scale level (Table 1, Figs. 3, 5 and Additional file 5). On the contrary, there were still a few diverged coding genes, including *matK*, *accD*, *psaJ*, *rpl16*, *ndhF*, *ycf1*, and so on. The *matK* and *ycf1* coding regions had been observed to be highly divergent and could serve as markers for DNA barcoding and phylogenetic analysis [32–35]. Similarly, nucleotide diversity analysis showed that eight genes (*trnK*, *matK*, *psaJ*, *rpl16*, *ndhF*, *ccsA*, *ndhA*, and *ycf1*) among eight Lardizabaloideae species had higher divergence values (Pi > 0.015), implying that they contained more variations than other coding genes (Fig. 6b). Among these genes, *matK*, *ndhF*, *ccsA*, and *ycf1* have been previously detected as highly variable regions in different plants, and some of those were served as DNA barcode [36–39]. However, previous studies confirmed that both introns and intergenic regions exhibited higher divergence levels than coding regions [40]. In our study, both genome-scale level alignments and nucleotide diversity analyses of the eight Lardizabaloideae chloroplast genomes revealed common variable sites, including eleven intergenic regions and eight coding genes (Figs. 5 and 6).

Previous studies supported that repetitive sequences were considered to play crucial roles in chloroplast genome arrangement and sequence divergence, even those were generally rare among angiosperm plastomes [41–43]. Generally, Lardizabaloideae species exhibited a significant difference in number and length of repeats within their chloroplast genomes. Most of the repeats were distributed in non-coding regions, including the intergenic regions and introns, reflecting the fact that the evolution of non-coding regions was higher than that of coding regions (Fig. 3) [44]. However, several repeats occurred in the same gene (*ycf2*) or paralogs (*pasA/psaB* and *trnS-GCU/trnS-UGA/trnS-GGA*), which might be caused by replication slippage, generating improper sequence recombination [45, 46]. Because of analytical and highly polymorphic nature, SSRs were considered to be well suited to assessment of genetic diversity within species and their relatives [47, 48]. In summary, repetitive sequences present in chloroplast genomes could facilitate the species discrimination and act as tools for investigating levels of genetic diversity in subfamily Lardizabaloideae.

### The adaptive evolution and positive selection

The Ka/Ks ratios were important to deduce the evolutionary rates and understand the adaptive developments among species [49]. The pairwise Ka/Ks ratios among *Akebia trifoliata*, *Akebia quinata*, *Stauntonia chinensis*, and *Archakebia apetala* were far less than 1, suggesting more intense purifying selection in these species, for both conservative and radical nonsynonymous substitutions (Fig. 7, Additional file 3). The lower Ka/Ks ratios might be explained that most genes in these species were likely to undergo deleterious nonsynonymous substitutions, and the purifying selection with stronger selective constraints for nonsynonymous substitutions than for synonymous ones [50, 51]. However, the Ka/Ks ratio between *H. angustifolia* and *H. latifolia* was greater than 1, implying some chloroplast coding sites of these two species were under positive selection. It is possible that more unknown selective forces might have contributed to the elevated Ka/Ks ratios, and resulted in species divergence [52].

It was suggested that codon sites with higher posterior probability could be also considered as positively selected sites, and genes containing positively selected sites might be evolving under divergent selective pressures [53, 54]. Although pairwise Ka/Ks ratios showed most of the chloroplast genes of Ranunculales species experienced purifying or no selection pressures, at least 25 chloroplast protein coding genes were identified with significant posterior probabilities suggesting sites with positive selection in Lardizabaloideae species, which indicated these genes might have evolved to adapt to environmental conditions (Table 5). Notably, we found that five of these 25 genes were associated with photosystem I and II subunits (*psaA*, *psaB*, *psaI*, *psbA*, and *psbZ*), while six of ten NADH-dehydrogenase subunit genes (*ndhD*, *ndhF*, *ndhH*, *ndhI*, *ndhJ*, and *ndhK*) possessed at least one positively selected site, implying these family members were potentially under positive selective pressure in Lardizabaloideae species (Fig. 8). Photosystem subunits and NADH-dehydrogenase subunits were essential in light energy utilization and electron transport chain for generation of ATP, which were all important components for photosynthesis of plants [55, 56]. Therefore, all these genes, which were involved in important process for plant growth and development, might evolve results of more frequent substitutions to adapt to different environmental conditions.

Among all positively selected genes, we found that the *accD* gene possessed the maximum number of sites under positive selection in Lardizabaloideae species, suggesting that the *accD* gene may play a pivotal role in the adaptive evolution of these species [57]. In addition, the likelihood ratio tests (LRTs) results showed that *p*-value of *rbcL* gene was less than 0.05, corroborating that sites in rubisco large subunit protein in clade Lardizabaloideae species have been under positive selection. As an important modulator of photosynthetic electron transport, recent study has revealed that positive selection of the *rbcL* gene was fairly common in all the main lineages of land plants [58, 59]. Thus, the *rbcL* gene was widely used to establish the diverse phylogenetic relationships of land plants [18, 60]. In summary, positive selection would possibly contribute to subfamily Lardizabaloideae diversification and adaptation.

### The phylogenetic analysis in order Ranunculales

Chloroplast genome sequences which contained sufficient information have been widely used to reconstruct phylogenetic relationships among angiosperms even at lower taxonomic levels [61–64]. The phylogenetic relationships based on CCG dataset were consistent with the Angiosperm Phylogeny Group (APG) IV system of classification [19]. Unexpectedly, the phylogenetic relationships based on both CCG and concatenated protein-coding genes CDs datasets were inconsistent. The phylogenetic tree based on CDs dataset showed that the family Circaeasteraceae species were clustered into a clade with family Ranunculaceae species. This result indicates that species *Kingdnia uniflora* and *Circaeaster agrestis* in family Circaeasteraceae had strong support to be a sister to the *Pulsatilla chinensis* in family Ranunculaceae based on chloroplast protein-coding genes, which was inconsistent with the APG IV classification system. The inconsistent phylogenetic relationships implied a different rate of evolution in coding regions and non-coding regions, which might due to the nucleotide substitutions of non-coding regions were noisy than those.

## Conclusions

This is the first report of the complete chloroplast genome sequence of *Stauntonia chinensis*. The architectural and the phylogenomic analysis of complete chloroplast genomes of eight Lardizabaloideae plants and relevant species could provide valuable genomic resource of this subfamily and its relatives. Meanwhile, several variation hotspots detected as highly variable regions could be served as the specific DNA barcodes. Our genomics analysis of these complete chloroplast genomes will lead to potential applications in the understanding of evolution and adaptation of species in the subfamily Lardizabaloideae.

## Methods

### Plant materials and DNA extraction

*Stauntonia chinensis,* which was identified by Prof. Liao Liang according to Flora of China, was sampled from Xianyan Mountain in Nanping city (118.10E, 26.73 N), Fujian Province, China. The voucher specimen deposited in Jiujiang University (accession number JJU130801). Approximately 5 g of fresh leaves was harvested for genomic DNA isolation using an improved extraction method [65].

### Chloroplast genome sequencing, assembly and annotation

A library with the insertion size of 430 bp was constructed, and all genome data were sequenced using an Illumina Hiseq 4000 platform at BIOZERON Co., Ltd. (Shanghai, China) [66]. The filtered reads were aligned with the *Akebia trifoliata* chloroplast genome (GenBank accession KU204898), and mapped to the reference chloroplast genomes [67, 68]. The chloroplast genes were annotated using an online DOGMA tool, using default parameters to predict protein-coding genes, transfer RNA (tRNA) genes, and ribosome RNA (rRNA) genes, coupled with manual check and adjustment [69].

### Codon usage, and repeat structure

Codon usage was determined for all protein-coding genes using the program Codon W 1.44 [70]. The relative synonymous codon usage (RSCU) was calculated to examine the deviation in synonymous codon usage. Six values were used to estimate the extent of bias toward codons: the codon adaptation index (CAI), codon bias index (CBI), frequency of optimal codons (Fop), the effective number of codons (ENC), GC content (GC), and GC content of synonymous third codons positions (GC3s).

Repeat structures (forward, palindromic, complement, and reverse) within the chloroplast genomes were analyzed using REPuter (https://bibiserv.cebitec.uni-bielefeld.de/reputer/), with following parameters: minimal repeat size of 30 bp and hamming distance of three [71]. Tandem repeats were identified using the Tandem Repeats Finder 4.09 (http://tandem.bu.edu/trf/trf.html) with parameters being set as 2, 7, and 7 for alignment parameters match, mismatch, and indels, respectively [72]. The minimum alignments score and maximum period size were 50 and 500, respectively. Perl script MISA was used to determine single sequence repeats (SSRs) within these chloroplast genomes with parameters of mono-, di-, tri-, tetra-, penta-, and hexa-nucleotides being set as 10, 5, 4, 3, 3, and 3, respectively [73].

### Genome comparison and nucleotide divergence

Comparative chloroplast genomes of eight Lardizabaloideae species were carried out and visualized by using mVISTA online software (http://genome.lbl.gov/vista/index.shtml) [74] with the *A. trifoliata* as a reference [67]. The large structural changes such as gene order rearrangements, inversions, and insertions were identified using Mauve v2.4.0 with default settings [75]. The chloroplast genome borders were also analyzed to show the IR expansions and contractions. DNAsp v5.10 software was used to analyze the nucleotide diversity (Pi) and sequence polymorphism of Lardizabaloideae species [76].

### Species pairwise Ka/Ks ratios and positive selected analyses

The concatenated single-copy gene coding sequences (CDs) of all 39 taxa were extracted and aligned with ClustalW [77]. Pairwise Ka/Ks ratios of all species were calculated using KaKs Calculator v2.0 [78]. The positive selected analyses were performed by an optimized branch-site model and Bayesian Empirical Bayes (BEB) method [53, 54]. The single-copy CDs of protein-coding genes of all 39 taxa were extracted and their amino acid sequences were aligned with ClustalW. The branch-site model was performed to test for potential positive selection using the CODEML algorithm implemented in EasyCodeML [79, 80]. The ratio (ω) of nonsynonymous to synonymous substitution rates was used to determine the selective pressure. The positive selection, no selection and negative selection were indicated when the ratio ω > 1, ω = 1, and ω < 1, respectively [80–82]. The likelihood-ratio tests (LRT) were performed according to Lan et al. [83]. The BEB method was used to compute the posterior probabilities of amino acid residues to identify whether these residue sites had potentially evolved under selection [53].

### Phylogenetic analyses

The complete chloroplast genome (CCG) sequences and concatenated single-copy protein coding genes CDs of all 39 taxa were aligned using ClustalW. The phylogenetic analyses were carried out through maximum likelihood (ML) and Bayesian inference (BI) performed in IQ-TREE v1.6.1 and MrBayes 3.1.2, respectively [84, 85]. The best-fit models for both datasets were selected by MrModeltest v2.3. The Maximum likelihood analyses were conducted using IQ-TREE with 1000 bootstrap replicates. The BI analysis was run for 100,000 generations and sampled every 100 generations. The first 25% of the trees were discarded as burn-in, and the remaining trees were used to build a 50% majority-rule consensus tree.

## Supplementary Information

**Additional file 1.** The statistics of codon usage bisa in all 39 taxa used in this study.

**Additional file 2 **Plastome alignment of all 39 taxa in this study. Gene arrangement map was carried out with only one copy of the IR using Mauve v2.4.0 software. The *Akebia trifoliata* chloroplast genome is shown at top as the reference genome. Local collinear blocks are represented by blocks of the same color connected by lines.

**Additional file 3.** Summary of Pairwise KaKs ratios in Lardizabaloideae and other families.

**Additional file 4.** Partial alignment of amino acids sequences in the other 19 positively selected genes.

**Additional file 5.** Summary of complete chloroplast genomes of all 39 taxa in this study.

**Additional file 6 **The complete cp genome of *Stauntonia chinensis*.

## Data Availability

All data generated or analyzed during this study were included in this published article and the Additional files. The complete cp genome of *Stauntonia chinensis* was submitted to GenBank under the accession number MN401678, which could also be found in Additional file 6. All raw reads are available in the short sequence archive under accession no. PRJNA700993. All of the complete genome sequences used in this study were downloaded from NCBI (https://www.ncbi.nlm.nih.gov), and the accession numbers can be found in Additional file 5.
