# Supplementary material for: The complete chloroplast genome of Stauntonia chinensis and compared analysis revealed adaptive evolution of subfamily Lardizabaloideae species in China
Source: BMC Genomics. 2021 Mar 6;22:161. doi: 10.1186/s12864-021-07484-7 (PMC7937279; doi:10.1186/s12864-021-07484-7)
Supplement: Supplementary file 1 — Additional file 1. The statistics of codon usage bisa in all 39 taxa used in this study. [file 12864_2021_7484_MOESM1_ESM.docx]

Additional file 1 The statistics of codon usage bisa in all 39 taxa used in this study

| Family | Access No | T3s | C3s | A3s | G3s | CAI | CBI | Fop | Nc | GC3s | GC | L_sym | L_aa | Gravy | Aromo |
| --- | --- | --- | --- | --- | --- | --- | --- | --- | --- | --- | --- | --- | --- | --- | --- |
| Lardizabalaceae | KU204898 | 0.4554 | 0.1814 | 0.4204 | 0.19 | 0.167 | -0.096 | 0.357 | 50.79 | 0.285 | 0.389 | 25088 | 26169 | -0.10436 | 0.108296 |
|  | KX611091 | 0.4556 | 0.1812 | 0.4204 | 0.1903 | 0.167 | -0.097 | 0.356 | 50.8 | 0.285 | 0.389 | 25079 | 26161 | -0.10375 | 0.108635 |
|  | MK468518 | 0.4554 | 0.1812 | 0.4206 | 0.1901 | 0.167 | -0.096 | 0.357 | 50.78 | 0.285 | 0.389 | 25072 | 26154 | -0.1027 | 0.108626 |
|  | KY200671 | 0.4561 | 0.1806 | 0.4228 | 0.1877 | 0.166 | -0.101 | 0.354 | 50.63 | 0.283 | 0.388 | 25073 | 26164 | -0.10245 | 0.109272 |
|  | MN401677 | 0.455 | 0.1813 | 0.421 | 0.1905 | 0.167 | -0.096 | 0.357 | 50.81 | 0.285 | 0.389 | 25254 | 26343 | -0.10721 | 0.108454 |
|  | MH394378 | 0.4551 | 0.1812 | 0.421 | 0.1905 | 0.167 | -0.096 | 0.357 | 50.8 | 0.285 | 0.389 | 25254 | 26343 | -0.10704 | 0.108416 |
|  | MK533615 | 0.4581 | 0.1781 | 0.4234 | 0.1881 | 0.165 | -0.103 | 0.353 | 50.42 | 0.281 | 0.387 | 25065 | 26158 | -0.10087 | 0.108915 |
|  | MN401678 | 0.4556 | 0.1812 | 0.4204 | 0.1903 | 0.167 | -0.097 | 0.356 | 50.8 | 0.285 | 0.389 | 25079 | 26161 | -0.10375 | 0.108635 |
| Menispermaceae | MH298220 | 0.4569 | 0.1809 | 0.4222 | 0.1878 | 0.167 | -0.099 | 0.355 | 50.62 | 0.283 | 0.389 | 24861 | 25962 | -0.10618 | 0.108736 |
|  | KU204903 | 0.4555 | 0.1809 | 0.4206 | 0.1916 | 0.167 | -0.097 | 0.356 | 51.02 | 0.286 | 0.389 | 24842 | 25920 | -0.10127 | 0.110262 |
|  | MH577056 | 0.4559 | 0.1793 | 0.4111 | 0.1922 | 0.166 | -0.099 | 0.353 | 50.85 | 0.287 | 0.395 | 23038 | 24058 | -0.04788 | 0.108322 |
| Eupteleaceae | KU204900 | 0.4546 | 0.1808 | 0.4194 | 0.1926 | 0.167 | -0.1 | 0.354 | 50.81 | 0.287 | 0.39 | 25148 | 26234 | -0.11691 | 0.108295 |
| Ranunculaceae | MK569490 | 0.457 | 0.178 | 0.4226 | 0.1931 | 0.165 | -0.104 | 0.352 | 50.84 | 0.284 | 0.387 | 25092 | 26165 | -0.10995 | 0.109841 |
|  | MG001341 | 0.4599 | 0.1752 | 0.4296 | 0.1883 | 0.164 | -0.108 | 0.35 | 50.5 | 0.277 | 0.382 | 25648 | 26758 | -0.10799 | 0.110322 |
|  | KY562594 | 0.4645 | 0.1701 | 0.4247 | 0.1916 | 0.164 | -0.113 | 0.347 | 50.37 | 0.276 | 0.383 | 24947 | 26026 | -0.09068 | 0.111196 |
|  | KM206568 | 0.4572 | 0.1781 | 0.4219 | 0.1933 | 0.165 | -0.105 | 0.352 | 50.85 | 0.284 | 0.386 | 25034 | 26115 | -0.10425 | 0.110894 |
|  | KX752098 | 0.4592 | 0.1757 | 0.4289 | 0.1854 | 0.165 | -0.106 | 0.351 | 50.38 | 0.277 | 0.384 | 25112 | 26216 | -0.10753 | 0.110276 |
|  | MH142266 | 0.4484 | 0.1858 | 0.4148 | 0.1995 | 0.165 | -0.101 | 0.354 | 51.8 | 0.296 | 0.393 | 25089 | 26173 | -0.11055 | 0.109922 |
|  | MF155666 | 0.4629 | 0.173 | 0.4254 | 0.1874 | 0.166 | -0.105 | 0.351 | 50.12 | 0.276 | 0.384 | 24633 | 25723 | -0.10616 | 0.108813 |
|  | MK569469 | 0.4553 | 0.1783 | 0.4219 | 0.1947 | 0.165 | -0.104 | 0.352 | 51.01 | 0.285 | 0.388 | 25177 | 26264 | -0.11159 | 0.109656 |
|  | MG010811 | 0.4587 | 0.1768 | 0.4297 | 0.1873 | 0.165 | -0.106 | 0.352 | 50.62 | 0.278 | 0.383 | 26777 | 27941 | -0.11767 | 0.110876 |
|  | MH205608 | 0.464 | 0.173 | 0.4319 | 0.1841 | 0.165 | -0.107 | 0.351 | 50.16 | 0.272 | 0.38 | 26508 | 27662 | -0.11484 | 0.11044 |
|  | MK569474 | 0.4516 | 0.1848 | 0.414 | 0.1988 | 0.165 | -0.1 | 0.354 | 51.61 | 0.294 | 0.393 | 24178 | 25247 | -0.10507 | 0.110389 |
|  | MK569476 | 0.4611 | 0.1733 | 0.429 | 0.1852 | 0.165 | -0.106 | 0.351 | 50.27 | 0.275 | 0.385 | 25992 | 27126 | -0.10753 | 0.1096 |
|  | MK569478 | 0.4584 | 0.1764 | 0.4286 | 0.1859 | 0.165 | -0.104 | 0.352 | 50.38 | 0.278 | 0.385 | 25071 | 26169 | -0.11111 | 0.110207 |
|  | MG675223 | 0.4606 | 0.1755 | 0.4282 | 0.1866 | 0.165 | -0.105 | 0.352 | 50.42 | 0.277 | 0.384 | 25875 | 26994 | -0.11736 | 0.109543 |
|  | KY120323 | 0.4557 | 0.1796 | 0.4184 | 0.1925 | 0.166 | -0.099 | 0.355 | 50.85 | 0.286 | 0.391 | 22797 | 23797 | -0.08599 | 0.109047 |
|  | KT964697 | 0.4627 | 0.1731 | 0.4249 | 0.1889 | 0.165 | -0.106 | 0.351 | 50.3 | 0.277 | 0.385 | 24561 | 25638 | -0.11108 | 0.109252 |
|  | KY085918 | 0.459 | 0.1776 | 0.4239 | 0.1901 | 0.166 | -0.106 | 0.352 | 50.67 | 0.282 | 0.388 | 24241 | 25280 | -0.14431 | 0.109415 |
|  | FJ597983 | 0.4598 | 0.1758 | 0.4283 | 0.1853 | 0.165 | -0.106 | 0.351 | 50.34 | 0.277 | 0.384 | 25061 | 26162 | -0.10841 | 0.110236 |
|  | MG675224 | 0.4613 | 0.1743 | 0.4294 | 0.1865 | 0.165 | -0.105 | 0.352 | 50.36 | 0.276 | 0.383 | 26017 | 27146 | -0.11927 | 0.109961 |
| Berberidaceae | KM057374 | 0.4537 | 0.1785 | 0.4219 | 0.1859 | 0.164 | -0.101 | 0.352 | 50.63 | 0.281 | 0.391 | 23132 | 24127 | -0.04876 | 0.108633 |
|  | MG234280 | 0.4589 | 0.1772 | 0.4164 | 0.1872 | 0.166 | -0.097 | 0.355 | 50.42 | 0.281 | 0.392 | 24443 | 25520 | -0.04948 | 0.109875 |
| Circaeasteraceae | KY908400 | 0.466 | 0.1725 | 0.4233 | 0.1838 | 0.165 | -0.103 | 0.351 | 50.17 | 0.274 | 0.386 | 23982 | 25026 | -0.08244 | 0.109166 |
|  | KY908401 | 0.4658 | 0.1734 | 0.437 | 0.1827 | 0.168 | -0.098 | 0.357 | 49.88 | 0.271 | 0.38 | 22810 | 23758 | -0.22463 | 0.106659 |
| Papaveraceae | KT274030 | 0.455 | 0.181 | 0.4237 | 0.1907 | 0.166 | -0.1 | 0.354 | 50.84 | 0.285 | 0.388 | 25354 | 26473 | -0.12322 | 0.10879 |
|  | MH394383 | 0.457 | 0.1797 | 0.4233 | 0.1893 | 0.166 | -0.102 | 0.353 | 50.56 | 0.283 | 0.388 | 25204 | 26307 | -0.12096 | 0.108336 |
|  | MH394399 | 0.4596 | 0.179 | 0.4233 | 0.1873 | 0.167 | -0.101 | 0.354 | 50.66 | 0.281 | 0.386 | 25021 | 26117 | -0.10559 | 0.110732 |
|  | KU204905 | 0.459 | 0.1788 | 0.4232 | 0.187 | 0.167 | -0.1 | 0.354 | 50.61 | 0.281 | 0.387 | 24900 | 25993 | -0.10541 | 0.109453 |
