# Supplementary figures and images for: The complete chloroplast genome of Stauntonia chinensis and compared analysis revealed adaptive evolution of subfamily Lardizabaloideae species in China
Source: BMC Genomics. 2021 Mar 6;22:161. doi: 10.1186/s12864-021-07484-7 (PMC7937279; doi:10.1186/s12864-021-07484-7)

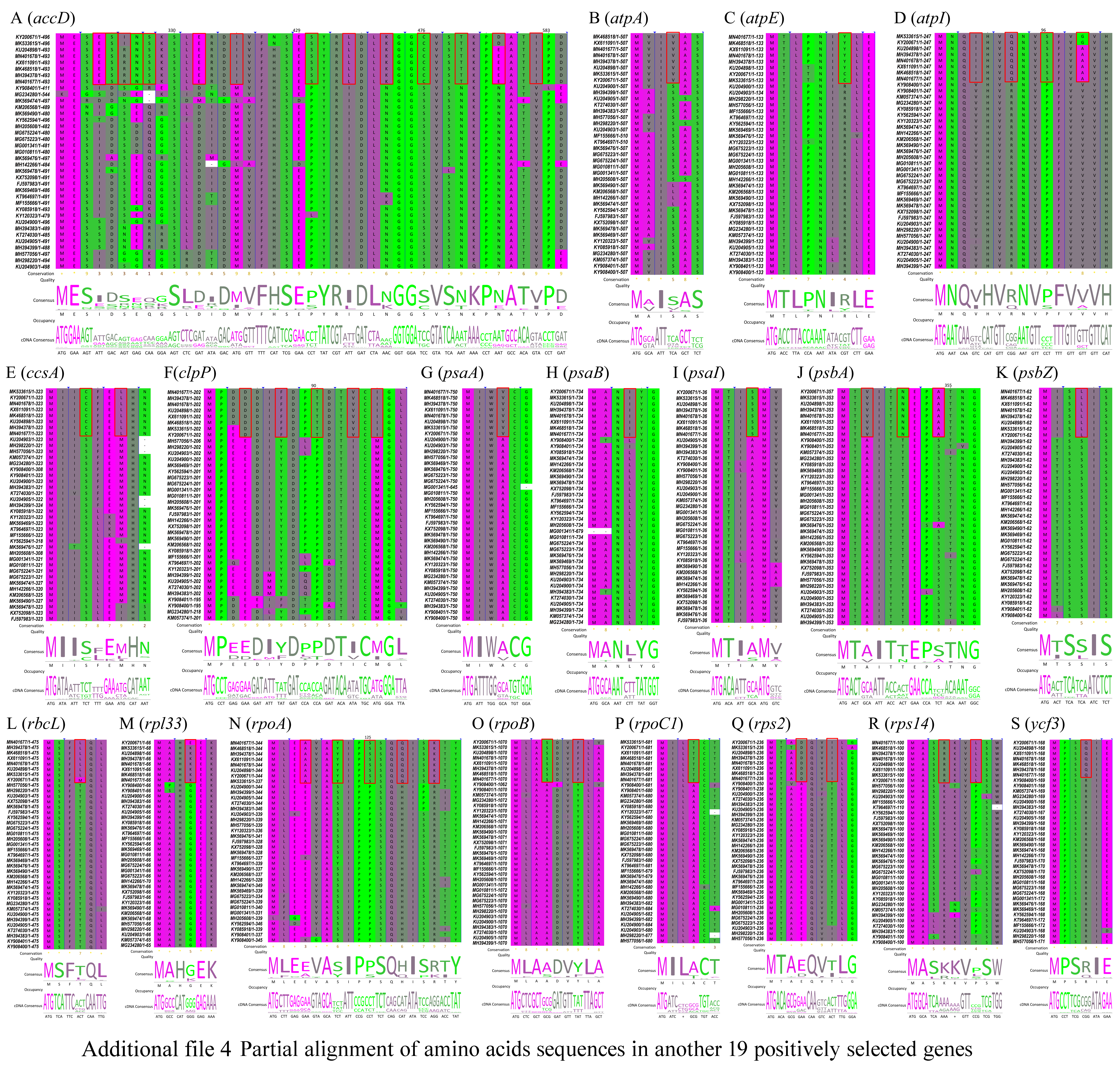

Supplement: Supplementary file 4 — Additional file 4. Partial alignment of amino acids sequences in the other 19 positively selected genes. [file 12864_2021_7484_MOESM4_ESM.png]
