# Supplementary material for: The complete chloroplast genome of Stauntonia chinensis and compared analysis revealed adaptive evolution of subfamily Lardizabaloideae species in China
Source: BMC Genomics. 2021 Mar 6;22:161. doi: 10.1186/s12864-021-07484-7 (PMC7937279; doi:10.1186/s12864-021-07484-7)
Supplement: Supplementary file 5 — Additional file 5. Summary of complete chloroplast genomes of all 39 taxa in this study. [file 12864_2021_7484_MOESM5_ESM.docx]

Additional file 5 Summary of complete chloroplast genomes of all 39 taxa used in this study

| Family | Species | Access No | Genome length (bp) | GC content （%） | LSC length (bp) | SSC length (bp) | IR length (bp) | Gene Number | Protein coding | tRNAs | rRNAs | No. of pseudogenes | GC3s content （%） |
| --- | --- | --- | --- | --- | --- | --- | --- | --- | --- | --- | --- | --- | --- |
| Lardizabalaceae | *Akebia trifoliata* | KU204898 | 158339 | 38.7 | 87057 | 19024 | 26129 | 132 | 85 | 37 | 8 | 2 | 28.5 |
|  | *Akebia quinata* | KX611091 | 157817 | 38.7 | 86543 | 18988 | 26143 | 132 | 85 | 37 | 8 | 2 | 28.5 |
|  | *Archakebia apetala* | MK468518 | 157929 | 38.7 | 86630 | 19001 | 26149 | 132 | 85 | 37 | 8 | 2 | 28.5 |
|  | *Decaisnea insignis* | KY200671 | 158683 | 38.5 | 87187 | 19162 | 26167 | 132 | 85 | 37 | 8 | 2 | 28.3 |
|  | *Holboellia angustifolia* | MN401677 | 157797 | 38.7 | 86543 | 18972 | 26141 | 132 | 85 | 37 | 8 | 2 | 28.5 |
|  | *Holboellia latifolia* | MH394378 | 157818 | 38.7 | 86567 | 18971 | 26140 | 132 | 85 | 37 | 8 | 2 | 28.5 |
|  | *Sinofranchetia chinensis* | MK533615 | 158015 | 38.4 | 86324 | 18923 | 26384 | 133 | 85 | 38 | 8 | 2 | 28.1 |
|  | *Stauntonia chinensis* | MN401678 | 157819 | 38.7 | 86545 | 18988 | 26143 | 132 | 85 | 37 | 8 | 2 | 28.5 |
| Menispermaceae | *Menispermum dauricum* | MH298220 | 158623 | 38.0 | 88879 | 20644 | 24550 | 132 | 85 | 37 | 8 | 2 | 28.3 |
|  | *Stephania japonica* | KU204903 | 157719 | 38.2 | 88693 | 20346 | 24340 | 132 | 85 | 37 | 8 | 2 | 28.6 |
|  | *Tinospora cordifolia* | MH577056 | 150945 | 38.7 | 83883 | 18782 | 24140 | 123 | 85 | 29 | 7 | 2 | 28.7 |
| Eupteleaceae | *Euptelea pleiosperma* | KU204900 | 161834 | 38.6 | 90449 | 19311 | 26037 | 132 | 85 | 37 | 8 | 2 | 28.7 |
| Ranunculaceae | *Paraquilegia anemonoides* | MK569490 | 164383 | 38.9 | 84925 | 17500 | 30979 | 132 | 83 | 37 | 8 | 4 | 28.4 |
|  | *Pulsatilla chinensis* | MG001341 | 162052 | 37.5 | 81923 | 17787 | 31171 | 134 | 89 | 36 | 8 | 1 | 27.7 |
|  | *Ranunculus repens* | KY562594 | 154247 | 37.9 | 84225 | 18434 | 25794 | 132 | 85 | 39 | 8 | 0 | 27.6 |
|  | *Thalictrum coreanum* | KM206568 | 155088 | 38.4 | 84733 | 17549 | 26403 | 132 | 83 | 37 | 8 | 4 | 28.4 |
|  | *Trollius chinensis* | KX752098 | 160191 | 38.1 | 88522 | 18405 | 26632 | 131 | 85 | 36 | 8 | 2 | 27.7 |
|  | *Urophysa henryi* | MH142266 | 158303 | 38.8 | 87031 | 18260 | 26506 | 131 | 85 | 36 | 8 | 2 | 29.6 |
|  | *Aconitum sinomontanum* | MF155666 | 157215 | 38.0 | 88074 | 16961 | 26090 | 128 | 82 | 38 | 8 | 0 | 27.6 |
|  | *Actaea asiatica* | MK569469 | 159638 | 38.1 | 88791 | 17711 | 26568 | 131 | 84 | 37 | 8 | 2 | 28.5 |
|  | *Anemoclema glaucifolium* | MG010811 | 160400 | 37.9 | 80251 | 17637 | 31256 | 139 | 94 | 36 | 8 | 1 | 27.8 |
|  | *Anemone trullifolia* | MH205608 | 157096 | 37.6 | 78795 | 16257 | 31022 | 136 | 92 | 36 | 8 | 0 | 27.2 |
|  | *Aquilegia coerulea* | MK569474 | 161429 | 39.0 | 91119 | 17354 | 26478 | 140 | 81 | 45 | 8 | 6 | 29.4 |
|  | *Asteropyrum cavaleriei* | MK569476 | 163660 | 38.0 | 82938 | 16896 | 31913 | 140 | 92 | 37 | 8 | 3 | 27.5 |
|  | *Calathodes oxycarpa* | MK569478 | 160415 | 38.0 | 89017 | 18404 | 26497 | 132 | 85 | 37 | 8 | 2 | 27.8 |
|  | *Clematis brevicaudata* | MG675223 | 159583 | 38.0 | 79339 | 18104 | 31070 | 134 | 89 | 36 | 8 | 1 | 27.7 |
|  | *Coptis chinensis* | KY120323 | 155484 | 38.2 | 84567 | 17393 | 26762 | 128 | 82 | 36 | 8 | 2 | 28.6 |
|  | *Gymnaconitum gymnandrum* | KT964697 | 157327 | 38.1 | 88107 | 16940 | 26140 | 129 | 83 | 37 | 8 | 1 | 27.7 |
|  | *Hydrastis canadensis* | KY085918 | 160000 | 38.6 | 87132 | 18804 | 27032 | 129 | 83 | 36 | 8 | 2 | 28.2 |
|  | *Megaleranthis saniculifolia* | FJ597983 | 159924 | 38.0 | 88326 | 18382 | 26608 | 131 | 85 | 36 | 8 | 2 | 27.7 |
|  | *Naravelia zeylanica* | MG675224 | 159568 | 37.9 | 79376 | 18086 | 31053 | 137 | 91 | 36 | 8 | 2 | 27.6 |
| Berberidaceae | *Berberis amurensis* | KM057374 | 166636 | 38.0 | 73665 | 18667 | 37152 | 140 | 94 | 38 | 8 | 0 | 28.1 |
|  | *Ranzania japonica* | MG234280 | 169224 | 38.0 | 74477 | 18899 | 37924 | 145 | 93 | 37 | 8 | 7 | 28.1 |
| Circaeasteraceae | *Circaeaster agrestis* | KY908400 | 151033 | 38.2 | 78130 | 16857 | 28023 | 133 | 85 | 38 | 8 | 2 | 27.4 |
|  | *Kingdonia uniflora* | KY908401 | 147378 | 37.8 | 80689 | 4857 | 30917 | 130 | 78 | 37 | 8 | 7 | 27.1 |
| Papaveraceae | *Coreanomecon hylomeconoides* | KT274030 | 158824 | 38.7 | 86916 | 18538 | 26685 | 134 | 87 | 37 | 8 | 2 | 28.5 |
|  | *Macleaya microcarpa* | MH394383 | 161118 | 38.6 | 88460 | 19050 | 26804 | 130 | 85 | 37 | 6 | 2 | 28.3 |
|  | *Meconopsis racemosa* | MH394399 | 153763 | 38.8 | 83857 | 17916 | 25995 | 129 | 84 | 37 | 6 | 2 | 28.1 |
|  | *Papaver somniferum* | KU204905 | 152931 | 38.9 | 83029 | 17920 | 25991 | 132 | 85 | 37 | 8 | 2 | 28.1 |
